# Case Report: An occurrence of steinstrasse in retrograde intra renal surgery (RIRS) for large staghorn kidney stone: a difficult experience in managing surgical outcomes

**DOI:** 10.12688/f1000research.22448.2

**Published:** 2020-05-29

**Authors:** Ponco Birowo, Nur Rasyid, Widi Atmoko, Bobby Sutojo

**Affiliations:** 1 Department of Urology, Faculty of Medicine Universitas Indonesia - Cipto Mangunkusumo Hospital, Jakarta Pusat, DKI Jakarta, 10430, Indonesia

**Keywords:** retrograde intrarenal surgery, staghorn stones, steinstrasse, complication

## Abstract

Immediate removal of staghorn kidney stones is important to prevent life-threatening complications. With the advancement of endoscopic technology, retrograde intrarenal surgery (RIRS) is now an alternate treatment to the standard percutaneous nephrolithotomy (PCNL) for stones removal. However, when used to treat large stones (>3cm), RIRS can cause the formation steinstrasse (SS). Here, we present the case of a 68-year-old man with multiple stones in the collecting system of the right kidney after initial treatment with RIRS. After two years of multiple interventions, the SS was completely removed. To prevent this complication in patients, a detailed assessment of the stone (size, location) and renal anatomy should be completed before RIRS is performed.

## Introduction

The term “staghorn” describes the configuration of large, branched renal stones that occupy the pelvis and extend to at least two renal calyces. Immediate removal of the stones is compulsory to prevent serious kidney injury and life-threatening sepsis ^1^. According to the American Urological Association, percutaneous nephrolithotomy (PCNL) is the standard treatment for staghorn removal ^2^. Recently, urologists have started using retrograde intrarenal surgery (RIRS) to treat large stones as it is less invasive and simpler than PCNL ^3^. However, RIRS might cause the formation of steinstrasse (SS), especially in large stones (2–3 cm) cases, which requires a series of interventions. This multiple procedure approach to renal stone treatment can impact patient quality of life, especially when the stone is hard (> 1000 Hounsfield Units) ^4, 5^.

The aim of this study is to address the formation of SS and the impact of prolonged treatment on the patient’s psychological health following the use of RIRS for large staghorn stone removal.

## Case presentation

A 68-year-old man came to our hospital in April 2016 with multiple stones in the collecting system of his right kidney. He had been experiencing flank pain that was not influenced by body position for one month. He denied any treatment relating to the pain that he experienced in this period. He also denied having a family history of this symptom or ever having this symptom before. Physical examination revealed only right flank tenderness.

**Figure 1.**
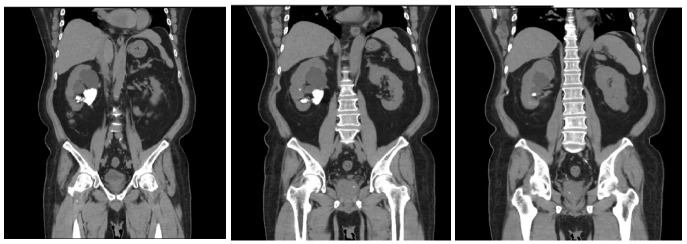
Initial computed tomography (CT) urography. The first CT Urography of the patient shows right staghorn stone with grade 3 hydronephrosis and left kidney cyst.

**Figure 2.**
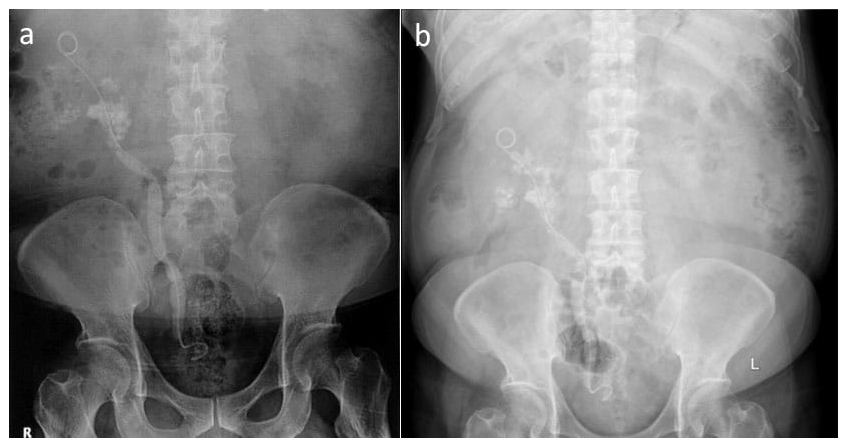
Steinstrasse formation. Immediate ( **a**) and one-month (April 2016) ( **b**) Kidney-Ureter-Bladder imaging following retrograde intrarenal surgery shows the right urinary system with multiple tiny stones.

Computed tomography (CT) urography at the previous hospital showed a staghorn stone at the right inferior calyx with a size of 45.7 × 59.3 × 27.5 mm (stone hardness in Hounsfield unit was not available) with a grade 3 right-side hydronephrosis and left kidney cyst. ( Figure 1). Post-RIRS imaging showed a double J (DJ) stent with multiple tiny stones from the right pelvio-calyces to vesicoureteral junction ( Figure 2a).

**Figure 3.**
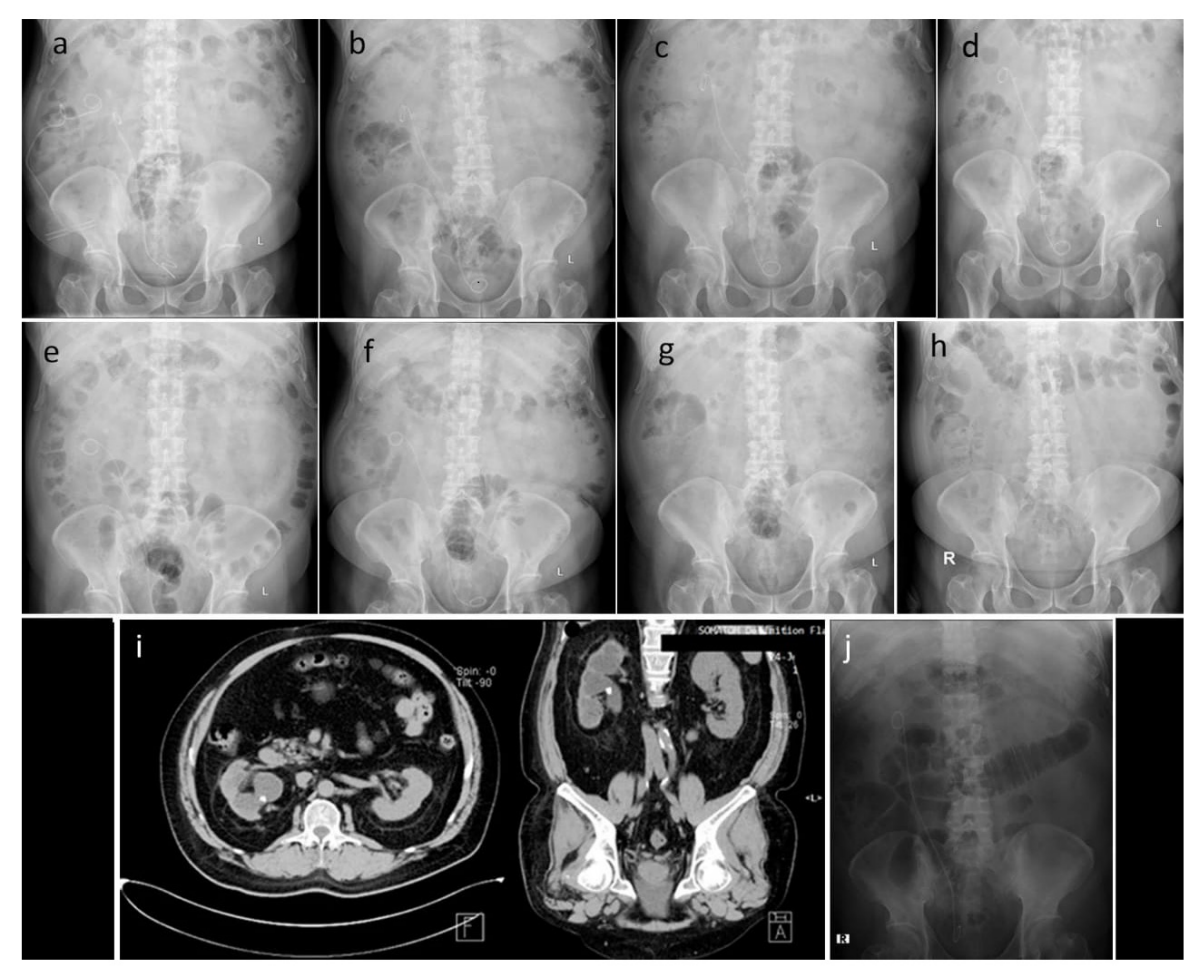
Sequential imaging photos. Imaging after right ureterorenoscopy, right nephrostomy, and right percutaneous nephrolithotomy in April 2016 ( **a**), imaging after ureterorenoscopy and percutaneous nephrolithotomy in June 2016 ( **b**), imaging after the second extracorporeal shock wave lithotripsy in June 2016 ( **c**), imaging after the third extracorporeal shock wave lithotripsy in July 2016 ( **d**), imaging after right laser ureterorenoscopy and replacement of right double J stent in July 2016 ( **e**), imaging after extracorporeal shock wave lithotripsy in October 2016 ( **f**), imaging after double J stent removal in October 2016 ( **g**), imaging as a routine control in January 2018 ( **h** & **i**), imaging after retrograde intrarenal surgery which shows no residual stone in June 2019 ( **j**).

A month later, when the patient came to our hospital for a second opinion, his kidney-ureter-bladder (KUB) imaging result had not changed ( Figure 2b). Right ureteroscopy (URS), right nephrostomy, and right PCNL were performed and post-operative KUB imaging was conducted ( Figure 3a). Another right URS was performed two weeks later, showing the remaining 8-mm stone at the ureter-pelvic junction (UPJ; Figure 3b).

Extracorporeal shock wave lithotripsy (ESWL) had been performed twice in June 2016, resulting in a decrease stone size to 6 mm. ( Figure 3c). Another ESWL was performed the next month ( Figure 3d). In July 2016, the patient underwent a right laser URS followed by replacement of the DJ stent ( Figure 3e). Three months later, another ESWL was performed ( Figure 3f). Shortly after, the remaining DJ stent was removed. KUB imaging still showed residual right nephrolithiasis. ( Figure 3g). In 2017, the patient presented with significant depression that he attributed to the numerous procedures, and he decided to end the treatment for his remaining stone. He reported a lack of spirit throughout the day since the failure of the last ESWL procedure and had a feeling that this stone would never be adequately treated, and his constant need for pain medication would continue.

Almost two years later (January 2018), routine KUB imaging and CT urography showed no change in his right nephrolithiasis ( Figure 3h, i). In June 2019, he was persuaded by his family to re-try stone management and had the final RIRS at another hospital, with successful complete removal of the remaining stone( Figure 3j). In November 2019, he visited our hospital for DJ stent removal. Neither stone nor DJ stent were observed in his KUB imaging. The summary of the patient’s history of illness is presented in Table 1.

**Table 1.** Summary of the patient’s history of illness.

Time	Initial condition	Procedure	Result
December 2015	KUB Imaging showed right staghorn stone (45.7 × 59.3 × 27.5 mm); Grade 3 hydronephrosis	RIRS and DJ stent insertion	Multiple tiny stones along the right urinary system from pelvio-calyces to vesico-ureteral junction ( Figure 2)
April 2016	KUB imaging showed multiple tiny stones along the right urinary system from pelvio-calyces to vesico-ureteral junction	Right URS; Right nephrostomy; Right PCNL; Insertion of a new DJ stent	A remaining radio opaque stone with a diameter of 8 mm at the ureteropelvic junction ( Figure 3a)
June 2016	KUB imaging showed an 8 mm radio opaque stone	ESWL twice	The stone size was decreased to 6 mm ( Figure 3b, c)
July 2016	KUB imaging showed a 6 mm radio opaque stone	ESWL; DJ stent replacement; Right laser URS	Small residual stones at the right kidney ( Figure 3d, e)
October 2016	A residual right nephrolithiasis	ESWL; DJ stent removal	A residual right nephrolithiasis ( Figure 3f)
January 2018	KUB imaging and CT urography showed right nephrolithiasis	N/A	Figure 3g
June 2019	CT urography showed right nephrolithiasis	RIRS and DJ stent insertion	Right DJ stent *in situ*; No residual stone ( Figure 3h, i)
November 2019	Right DJ stent *in situ*; No residual stone	DJ stent removal	No stone was found on the final KUB imaging ( Figure 3j)

CT, computed tomography; DJ, double J; ESWL, extracorporeal shock wave lithotripsy; KUB, kidney-ureter-bladder; PCNL, percutaneous nephrolithotomy; RIRS, retrograde intrarenal surgery; URS, Ureteroscopy.

## Discussion

The management of nephrolithiasis has changed dramatically over time, shifting from open surgery to less-invasive procedures, such as PCNL and ESWL ^6^. According to the American Urological Association and European Association of Urology guidelines, the standard treatment for staghorn stone removal is PCNL ^2, 5^. PCNL has a high stone-free rate (SFR), similar to that of an open surgery (93%). It also results in lower morbidity, shorter operative time, shorter hospital stays, and earlier back to work compared to open surgery. However, it can cause severe complications, such as renal trauma with severe uncontrollable bleeding ^7, 8^.

On the other hand, the development of flexible ureteroscopes allows for excellent visualization that makes RIRS a favourable procedure for most urologists. The possibility to use holmium lasers along with the ureteroscope, and lower cost compared to the other treatment methods, has made this procedure even more popular ^9^. Initially, the use of RIRS is limited to patients who cannot undergo PCNL or ESWL due to several contraindications. However, with the development of technology, the usage of RIRS for large stone is now possible. Compared to PCNL, RIRS has a slightly lower SFR of 87% and also lower morbidity and complication rate of 2% ^10^. In our case, use of RIRS instead of PCNL as the first treatment was due to the patient’s preference for a less invasive method.

RIRS is a less-invasive procedure compare to PCNL. Complications may arise intra- or post-operatively in some cases but are usually minor and manageable. The common complications of RIRS include hemorrhage, intrapelvic hematoma, mucosal injury, ureteral perforation and avulsion, urinary tract infection, and sepsis ^11^. In a study by Niwa *et al*., the most common complication associated with RIRS in treating staghorn stones was urinary tract infection (Clavien-Dindo II, 28.2%), followed by fever (7.7%), general malaise (2.6%), and malposition of a ureteral stent (2.6%) ^12^.

In Indonesia, PCNL is still the first choice for treating large renal calculi according to *Ikatan Ahli Urologi Indonesia* (the Indonesian Urologist Association). However, the use of PCNL in Indonesia is still limited due to the lack of technology and expertise, particularly in remote areas ^13^. The incidence of SS formation after RIRS is 20% among those with large renal stone, while hydronephrosis is also common ^4^. The development of SS was also observed in the patient we have described, who was initially treated with RIRS. To address this complication, a scoring system was developed by Resorlu *et al*. that includes four indicators: a renal stone size >20 mm, lower pole stone with an infundibulum-pelvic angle <45°, a stone number in different calyces >1, and abnormal renal anatomy ^14^. A greater score is associated with a lower SFR. This score can be calculated prior to RIRS.

Another efficacy parameter for RIRS is stone composition. According to a study by Xue *et al*., stones that are made of calcium oxalate dihydrate, uric acid, and magnesium ammonium phosphate show an excellent response to RIRS treatment ^15^. Unfortunately, in the present case, the stone composition was not analyzed due to financial constraints.

In the previous hospital, the ureteral stent was placed after RIRS treatment. The necessity for routine stent insertion before or after RIRS to increase stone clearance remains unclear. The primary purpose of stent insertion is to prevent ureteral stricture, accelerate healing, and facilitate stone passing ^16^. On the other hand, stent insertion increases the possibility of urinary tract infection, dysuria, pollakiuria, hematuria, and may require repeated cystoscopy in cases of stent migration and need for extraction ^17^. Stent insertion before ESWL does not eliminate the need for intervention in the management of SS ^18^. In cases like the one we have presented, considering the size and the position of the stone, ureteral stent placement before RIRS would be difficult and other options should be considered.

Urolithiasis is a painful chronic disease that has significantly impacts on a patient’s quality of life. In addition to chronic pain, the acute pain of urolithiasis resulting from stone movement often causes fear of recurrence. Recent studies have suggested an association between the disease and anxiety and depression ^19^. In the present study, our patient developed symptoms of depression during the second year of his treatment because he had to undergo multiple surgical procedures within a year to remove the SS. In addition, the patient had to endure the pain associated with recovery after each procedure, as well as the pain caused by the remaining stone. After receiving support from his family and reassurance by clinicians, the patient was finally convinced to continue with treatment for his remaining stones.

RIRS may be used in cases where open surgery and ESWL are risky or inadequate, such as in patients with obesity, bleeding disorders, musculoskeletal deformities, renoureteral malformations, and infundibular stenosis ^16^.

This study was limited in that we did not know the hardness (Hounsfield units) of the patient’s stone before he visited our clinic; therefore, we could not more precisely determine the cause of his previous treatment failure, as our characterization was based only on the size of the stone.

## Conclusions

RIRS is not the preferred option for removal of large staghorn calculi due to low efficacy and other possible complications. However, it can be used in circumstances where open surgery or PCNL are not possible. Careful assessment is essential to determine whether the procedure will be beneficial and safe for the patient.

## Data availability

All data underlying the result are available as part of the article and no additional source data are required.

## Consent

Written informed consent for publication of their clinical details and/or clinical images was obtained from the patient/parent/guardian/relative of the patient.
