# Supplementary material for: Case Report: An occurrence of steinstrasse in retrograde intra renal surgery (RIRS) for large staghorn kidney stone: a difficult experience in managing surgical outcomes
Source: F1000Res. 2020 May 29;9:184. Originally published 2020 Mar 12. [Version 2] doi: 10.12688/f1000research.22448.2 (PMC7338919; doi:10.12688/f1000research.22448.2)
Supplement: Peer review history

# Peer review: Reviewer response for version 2

**DOI:** 10.5256/f1000research.26885.r64109

**Published:** 2020-07-06

**Authors:** Hammad Ather

**Affiliations:** 1 Department of Surgery, Aga Khan University, Karachi, Pakistan

The authors have successfully incorporated the suggestions and modified the manuscript. I have no further observations on the current submission (V2).

Are enough details provided of any physical examination and diagnostic tests, treatment given and outcomes?

Yes

Is the case presented with sufficient detail to be useful for other practitioners?

Partly

Is sufficient discussion included of the importance of the findings and their relevance to future understanding of disease processes, diagnosis or treatment?

Yes

Is the background of the case’s history and progression described in sufficient detail?

No

Reviewer Expertise:

NA

I confirm that I have read this submission and believe that I have an appropriate level of expertise to confirm that it is of an acceptable scientific standard.

# Peer review: Reviewer response for version 2

**DOI:** 10.5256/f1000research.26885.r64110

**Published:** 2020-06-24

**Authors:** Doddy Soebadi

**Affiliations:** 1 Department of Urology, Faculty of Medicine, Airlangga University, Surabaya, Indonesia

The authors have revised the article from the first manuscript. The revision could be seen from the title and also in the introduction, discussion as well as the case presentation sections. We could see that the results are encouraging and make this article better in terms of the art of writing as well as from the scientific manuscript.

Are enough details provided of any physical examination and diagnostic tests, treatment given and outcomes?

Yes

Is the case presented with sufficient detail to be useful for other practitioners?

Yes

Is sufficient discussion included of the importance of the findings and their relevance to future understanding of disease processes, diagnosis or treatment?

Yes

Is the background of the case’s history and progression described in sufficient detail?

Yes

Reviewer Expertise:

General urology, voiding dysfunction, sexual medicine, endourology, laser urology.

I confirm that I have read this submission and believe that I have an appropriate level of expertise to confirm that it is of an acceptable scientific standard.

# Peer review: Reviewer response for version 1

**DOI:** 10.5256/f1000research.24773.r61757

**Published:** 2020-05-04

**Authors:** Hammad Ather

**Affiliations:** 1 Department of Surgery, Aga Khan University, Karachi, Pakistan

1. Steinstrasse is a legacy of SWL (Does ureteral stenting prior to shock wave lithotripsy influence the need for intervention in steinstrasse and related complications? (Ather *et al.*, 2009 ^1^)) and RIRS for larger stones is no different from performing SWL under endoscopy. The authors should discuss this point.
2. Conclusions are contrary to the case account. The conclusions should be that larger stones (Staghorn) would preferably not be treated by RIRS.
3. Few minor issues:

- In the abstract I don’t agree with the word “compulsory” - a more preferable alternative would be “desirable" or “important” etc.
- RIRS is not mentioned as a treatment option for large kidney stones.
- Mention other complications of the use of RIRS besides steinstrasse for large renal stones.
- The phrase “RIRS to remove a staghorn stone” in the abstract is not an appropriate reflection of the abilities of RIRS in that set up.
- There are many grammatical errors that need attention.
- The authors used the phrase “which led to depression in the patient after the use of RIRS for staghorn stone removal”. This needs to be rephrased for clarity as to what the authors are implying. Are they saying prolonged treatment causes psychological issues, like depression? Please cite a reference.
- In the case presentation, the authors write “multiple stones along his right urogenital system” - this needs to be rewritten for clarity. Stones were in the collecting system of the right kidney.
- With the limited access to single section I disagree with the authors' interpretation of the CT. I see a Staghorn stone with a major component in the renal pelvis and branching calculi in lower and middle pole calyces. There is dilatation and obstruction with ballooning of the upper pole calyx due obstruction of the infundibulum of the upper pole calyx. There is no cyst visible in these scans.
- It is grade three, not third grade hydronephrosis, which is incidentally also not clear in the CT cut shown.
- The authors should avoid using term “genitourinary”.
- What is the stone composition?

Are enough details provided of any physical examination and diagnostic tests, treatment given and outcomes?

Yes

Is the case presented with sufficient detail to be useful for other practitioners?

Partly

Is sufficient discussion included of the importance of the findings and their relevance to future understanding of disease processes, diagnosis or treatment?

Yes

Is the background of the case’s history and progression described in sufficient detail?

No

Reviewer Expertise:

Urolithiasis and Bladder cancer

I confirm that I have read this submission and believe that I have an appropriate level of expertise to confirm that it is of an acceptable scientific standard, however I have significant reservations, as outlined above.

## References

[1] : Does ureteral stenting prior to shock wave lithotripsy influence the need for intervention in steinstrasse and related complications?. *Urol Int*.2009;83(2) : 10.1159/000230028 222-5 10.1159/000230028 19752621

# Response

**Published:** 2020-05-23

**Authors:** Ponco Birowo

**Affiliations:** Department of Urology Faculty of Medicine Universitas Indonesia/ CIpto Mangunkusumo Hospital, Indonesia

**Comments from Reviewer and Author Response:**

 

- Steinstrasse is a legacy of SWL (Does ureteral stenting prior to shock wave lithotripsy influence the need for intervention in steinstrasse and related complications? (Ather et al., 2009 ^1^) and RIRS for larger stones is no different from performing SWL under endoscopy. The authors should discuss this point.

 

**Author response:**

We have revised and added this additional paragraph to our manuscript:

The necessity for routine stent insertion before or after RIRS to increase stone clearance remains unclear. The main purpose of stent insertion is to prevent ureteral stricture, accelerate healing, and facilitate stone passing (Cleynenbreugel, et al., 2017). On the other hand, stent insertion increases the possibility of urinary tract infection, dysuria, pollakiuria, hematuria, and repeated cystoscopy may be required in cases of stent migration and to assess the need for extraction (Ozyuvali, et al., 2015). Stent insertion before shockwave lithotripsy (SWL) does not eliminate the need for intervention in the management of steinstrasse (Ather et al., 2009). In the present case, considering the size and the position of the stone, ureteral stent placement prior to RIRS would be difficult and other options should be considered.

 

- Conclusions are contrary to the case account. The conclusions should be that larger stones (Staghorn) would preferably not be treated by RIRS.

 

**Author response:**

Thanks for your advice. We have revised our conclusion to become: RIRS is not the preferable option to remove large staghorn calculi due to lower efficacy and other possible complications. It can be used in circumstances where open surgery or PCNL is not possible, but careful assessment is necessary to determine whether the procedure will be beneficial and safe for the patient.

 

- In the abstract I don’t agree with the word “compulsory” - a more preferable alternative would be “desirable" or “important” etc.

 

**Author response:**

Thank you for your advice, we have changed the word according to your advice.

 

- RIRS is not mentioned as a treatment option for large kidney stones.

 

**Author response:**

We have revised and added this additional paragraph to our manuscript:

Initially, the use of RIRS is limited to patients who cannot undergo PCNL or shockwave lithotripsy (SWL) due to several contraindications. However, with the development of technology, the usage of RIRS for large stone is now possible. Compared to PCNL, RIRS has a slightly lower SFR of 87% and also lower morbidity and complication rate of 2%. ^10^ In our case, the use of RIRS instead of PCNL as the first treatment was due to the patient’s preference for a less invasive method.

 

- Mention other complications of the use of RIRS besides steinstrasse for large renal stones.

 

**Author response:**

We have revised and added this additional paragraph to our manuscript:

RIRS is a less-invasive procedure compared to PCNL. Complications may arise intra- or post-operatively in some cases but are usually minor and manageable. The common complications of RIRS include hemorrhage, intrapelvic hematoma, mucosal injury, ureteral perforation and avulsion, urinary tract infection, and sepsis. In a study by Niwa et al., the most common complication associated with RIRS in treating staghorn stones was urinary tract infection (Clavien-Dindo II, 28.2%), followed by fever (7.7%), general malaise (2.6%), and malposition of a ureteral stent (2.6%).

 

- The phrase “RIRS to remove a staghorn stone” in the abstract is not an appropriate reflection of the abilities of RIRS in that set up.

 

**Author response:**

We have revised our abstract as it is not aligned with the insight of our case presentation that do not recommend the use of RIRS for large stones removal. Here is the revised version:

Immediate removal of staghorn kidney stones is important to prevent life-threatening complications. With the advancement of endoscopic technology, retrograde intrarenal surgery (RIRS) is now an alternate treatment to the standard percutaneous nephrolithotomy (PCNL) for stones removal. However, when used to treat large stones (>3cm), RIRS can cause the formation steinstrasse (SS).

 

- There are many grammatical errors that need attention.

 

**Author response:**

Thank you for addressing your concern. We have revised the grammatical error that we found.

 

- The authors used the phrase “which led to depression in the patient after the use of RIRS for staghorn stone removal”. This needs to be rephrased for clarity as to what the authors are implying. Are they saying prolonged treatment causes psychological issues, like depression? Please cite a reference.

 

**Author response:**

We have added some information to clarify this issue.

Urolithiasis is a painful chronic disease that has significant impacts on a patient’s quality of life. In addition to chronic pain, the acute pain of urolithiasis resulting from stone movement often causes fear of recurrence. Recent studies have suggested an association between the disease and anxiety and depression. In the present study, our patient developed symptoms of depression during the second year of his treatment because he had to undergo multiple surgical procedures within a year to remove the SS. In addition, the patient had to endure the pain associated with recovery after each procedure, as well as the pain caused by the remaining stones.

 

- In the case presentation, the authors write “multiple stones along his right urogenital system” - this needs to be rewritten for clarity. Stones were in the collecting system of the right kidney.

 

**Author response:**

Thank you for your correction. We have updated the term accordingly.

 

- With the limited access to a single section I disagree with the authors' interpretation of the CT. I see a Staghorn stone with a major component in the renal pelvis and branching calculi in lower and middle pole calyces. There is dilatation and obstruction with ballooning of the upper pole calyx due to obstruction of the infundibulum of the upper pole calyx. There is no cyst visible in these scans.

 

**Author response:**

Thank you for your concern. We have decided to change the picture:

 

- It is grade three, not third-grade hydronephrosis, which is incidentally also not clear in the CT cut shown.

 

**Author response:**

We have changed “third grade” to “grade three” hydronephrosis.

 

- The authors should avoid using term “genitourinary”.

 

**Author response:**

Thank you. We have altered “genitourinary” to urinary system.

 

- What is the stone composition?

 

**Author response:**

Unfortunately, in the present case, the stone composition was not analyzed due to financial constraints.

 

- This is a good example of how minimally invasive surgeries can take a long journey before they come to a satisfactory result.

 

**Author response:**

Thank you for your appreciation.

 

- The authors did not mention the drawbacks of these lengthy managements, i.e. the opinion from the patient side and the cost of the serial treatments.

 

**Author response:**

The patient self-funded the whole treatment but he refused to tell the amount he had to pay. As for his opinion about the lengthy procedure, the patient complained of slight depression due to the continuous pain, either from the remaining stone and from the procedures. This information is mentioned in the case presentation and discussion section.

 

- But this is a very good clinical experience to be one of the considerations before the urologist(s) offer this minimally invasive procedure(s).

 

**Author response:**

Thank you. We hope this study provides a handful of insight regarding the disadvantage of RIRS to treat large kidney stone.

# Peer review: Reviewer response for version 1

**DOI:** 10.5256/f1000research.24773.r61758

**Published:** 2020-04-14

**Authors:** Doddy Soebadi

**Affiliations:** 1 Department of Urology, Faculty of Medicine, Airlangga University, Surabaya, Indonesia

- This is a good example of how minimally invasive surgeries can take a long journey before they come to a satisfactory result.
- The authors did not mention the drawbacks of these lengthy managements, i.e. the opinion from the patient side and the cost of the serial treatments.
- But this is a very good clinical experience to be one of the considerations before the urologist(s) offer this minimally invasive procedure(s).

Are enough details provided of any physical examination and diagnostic tests, treatment given and outcomes?

Yes

Is the case presented with sufficient detail to be useful for other practitioners?

Yes

Is sufficient discussion included of the importance of the findings and their relevance to future understanding of disease processes, diagnosis or treatment?

Yes

Is the background of the case’s history and progression described in sufficient detail?

Yes

Reviewer Expertise:

General urology, voiding dysfunction, sexual medicine, endourology, laser urology.

I confirm that I have read this submission and believe that I have an appropriate level of expertise to confirm that it is of an acceptable scientific standard.
